# Artificial Intelligence in the Management and Treatment of Pediatric Burns: A Narrative Review of Current Applications and Future Directions

**DOI:** 10.7759/cureus.98091

**Published:** 2025-11-29

**Authors:** Roban Shabbir, Emilia Preda, Harleen K Multani, Tioluwa Akinjaiyeju, Shaina Gagadam, Ayham Alkiswani, Julia Burger, MD

**Affiliations:** 1 Orthopaedics, Lewis Katz School of Medicine - Temple University, Philadelphia, USA; 2 Physiology, MedStar Georgetown University Hospital, Washington, USA; 3 Dermatology, Meharry Medical College School of Medicine, Nashville, USA; 4 Dermatology, Chobanian and Avedisian School of Medicine, Boston University, Boston, USA; 5 Dermatology, Philadelphia College of Osteopathic Medicine, Philadelphia, USA; 6 Dermatology, University of the Incarnate Word School of Osteopathic Medicine, San Antonio, USA; 7 Pediatrics, Lewis Katz School of Medicine - Temple University, Philadelphia, USA

**Keywords:** burn wound management, machine learning in healthcare, pediatric burns, predictive analytics & ai, telemedicine services, wearable sensors

## Abstract

Pediatric burn evaluation is often subjective; early estimates of depth and TBSA% drive triage, surgery, and follow-up. Emerging artificial intelligence (AI) tools, especially image-based methods, aim to standardize assessment and extend expertise via telemedicine.

To narratively review AI applications relevant to pediatric burn care across diagnosis (depth/healing), TBSA% estimation, treatment planning/monitoring, telemedicine, and rehabilitation, and to summarize practical and ethical considerations for translation.

Narrative review of PubMed/MEDLINE and Embase (January 2000-June 2025) with citation chaining. Pediatric and mixed-age studies applicable to pediatric care were included; conference abstracts and preprints were excluded unless subsequently published. Heterogeneity precluded quantitative synthesis; results are qualitatively summarized.

Evidence is strongest for image-based assessment (clinical photographs, multispectral/optical modalities) supporting depth/healing classification and TBSA% segmentation, with several studies showing promising discrimination compared with unaided clinical assessment. Telemedicine workflows for remote triage and follow-up appear feasible, while data-driven decision support for timing of surgery and advanced dressing strategies remains investigational. Pediatric-specific datasets, external validation against accepted comparators, and reporting across skin tones and ages are still limited.

AI has the potential to complement clinician judgment in pediatric burn care, most immediately for early image-based assessment and remote follow-up. Broader adoption will require pediatric-focused datasets, rigorous external validation, transparent governance for privacy and bias, and clear human-in-the-loop oversight.

## Introduction and background

Pediatric burns rank among the leading causes of morbidity and irreversible disability in children [1]. Common presentations include scalds, flame injuries, and contact with hot objects, which can subsequently lead to increased severity of complications such as infections, contractures, scarring, and psychosocial impacts. The unique physiological and emotional vulnerabilities of pediatric patients make these treatments more pressing. Pediatric burn care is complicated by the subjective clinical assessment of burn depth and the need for developmentally appropriate treatment strategies, pain management, and rehabilitation [2]. Physicians with less experience may make inaccurate assessments, further complicating diagnosis and long-term planning.

Patterns vary by region and home environment (i.e, scalds predominate in toddlers and younger children [1,3]. Children’s higher surface-area-to-mass ratio and immature thermoregulation confer greater risks of hypothermia and fluid shifts, while long-term sequelae include contractures, psychosocial distress, and excessive school absences [3]. Objective depth assessment is especially critical in pediatrics, where inaccurate early classification can delay surgery or overtreat superficial injuries. For example, Laser Doppler Imaging (LDI) has outperformed clinical exam for predicting outcomes in children [4-6]. Emerging artificial intelligence (AI) image-analysis and decision-support tools seek to standardize these assessments and extend specialist expertise via telehealth [7,8].

Two assessments largely determine downstream care: burn depth and percent total body surface area (TBSA%). Bedside depth assessment is inherently subjective and, when compared against outcome or histology, clinical accuracy often hovers near 60-70%, whereas LDI can achieve up to 97% accuracy in intermediate-depth burns [9-11]. Misclassification has consequences: overestimation may prompt unnecessary grafting; underestimation risks delayed excision, infection, and scar hypertrophy. These risks climb as time-to-healing exceeds 3 weeks [12,13]. These pitfalls are amplified in pediatrics, given distinct resuscitation and metabolic considerations [14]. 

AI has gained traction in various medical settings, with significant potential in image analysis, predictive modeling, and healthcare assessment and protocols [15]. Studies have demonstrated that computer vision-based models outperform experienced clinicians in classifying burn depth and predicting healing times, particularly in terms of speed and consistency [16]. AI further helps to delineate indications and guidelines for surgical planning and rehabilitation [17]. One such application within telemedicine is an integrated wearable sensor and remote monitoring system, which shows promise for post-discharge care in low-resource settings [18].

Recent work has expanded beyond clinical inspection and classic perfusion tools to include non-invasive imaging and AI methods that improve early classification and triage. Contemporary reviews summarize rapid progress in ML for optical or spectral burn imaging [19-21], while clinical studies report deep-learning models that segment burns, estimate TBSA, and classify surgical need, often using smartphone photographs or multispectral sensors [7,8,22-24]. Pediatric-focused advances (e.g., OCT scoring for children’s hand burns) further emphasize modality choices that could complement or benchmark emerging AI [25]. In parallel, meta-analytic evidence continues to anchor perfusion-based comparators such as LDI [26], supporting our decision to evaluate novel methods against established diagnostic references.

This review examines the current and future applications of AI in pediatric burn management. There are clinical challenges unique to children, where AI may play a crucial role in diagnosing, monitoring, and evaluating potential contributions to surgical decision-making and rehabilitation. With the surge of AI, the unique ethical considerations underscore the importance of integrating responsible AI for equitable and effective pediatric care.

## Review

Methods

Eligibility Criteria

Pediatric or mixed-age studies relevant to pediatric burn care that reported AI-enabled diagnosis (depth/healing), TBSA% estimation/segmentation, treatment planning, telemonitoring/wearables, or rehabilitation. We included peer-reviewed clinical studies, validation studies, and substantial engineering studies with clinical test sets; conference abstracts and preprints were excluded unless subsequently published. Studies were excluded if they did not include any burn wounds (i.e., pressure ulcers, diabetic foot), pediatric data, insufficient reporting/clinical data, or peer review. 

Information Sources

PubMed/MEDLINE and Embase (January 2000 - October 2025), plus manual citation chaining from identified papers.

Search Strategy

Combined terms for children/pediatrics, burns, and AI/ML (e.g., “(pediatric* OR child*) AND (burn* OR scald*) AND (artificial intelligence OR machine learning OR deep learning OR computer vision OR segmentation OR telemedicine OR wearable* OR rehabilitation)”). Filters: English language; no geographic limits.

Study Selection

Three authors independently screened titles/abstracts for relevance to pediatric burn applications; disagreements were resolved by discussion. A PRISMA flow diagram was not constructed as we did not intend a quantitative synthesis.

Data Items

We noted simple descriptors reported by each paper: study setting (e.g., inpatient, outpatient, telemedicine), pediatric age range, approximate sample size, and imaging modality (e.g., clinical photographs, multispectral/optical).

Risk-of-Bias

A formal risk-of-bias instrument was not completed; we qualitatively considered small samples, spectrum bias (adult-dominant cohorts), reference-standard quality, and data-splitting risks when interpreting results.

Synthesis Methods

Qualitative narrative synthesis only; no pooling or meta-analysis was planned, given the heterogeneity in imaging platforms, reference standards, and pediatric case mix.

Ethics

This narrative review used publicly available, published literature and did not involve human participants or identifiable data; institutional review board approval and consents were not required.

Review

Overview of Pediatric Burn Treatment

When diagnosing a pediatric burn injury, the depth of tissue damage and the TBSA% affected are integral parts of the classification system. They dictate clinical management and fluid resuscitation to alleviate the secondary hypovolemia caused by shifts in fluid following the injury [27]. Burn classification is determined by clinical evaluation of many factors, including appearance, layer of skin affected, and pain assessment. Superficial or first-degree burns commonly involve the epidermis, the outermost layer of the skin. Superficial burns are characterized by redness and pain, but they typically heal within a week with no long-term effects [28]. Second-degree, or partial-thickness, burns penetrate deeper into the dermis, appearing moist, soft, and very painful. Scald burns, especially those caused by hot liquids, account for nearly ⅓ of burns in children and are often associated with partial-thickness burns [29]. The prevalence of scald burns highlights the importance of early identification and classification to prevent complications. Third-degree burns usually destroy all layers of the dermis and penetrate the subcutaneous tissue, and the patient's skin appears to be charred or leathery in texture [28]. 

Wound management involves gentle debridement and the topical application of antimicrobial agents, such as silver sulfadiazine, to prevent infection [30]. Adequate pain control is particularly critical in the pediatric patient population since ineffective pain control may result in chronic psychological distress. Pain relief methods include pharmacological treatment and other remedies, such as distraction or relaxation techniques to ease the mind and body [31]. Patients with more severe burn injuries undergo surgical debridement to remove dead skin and tissue, along with skin grafting, to provide expedited recovery and minimize the risk of infection [32]. Rehabilitation is initiated early and sustained throughout recovery, aiming to strengthen mobility, prevent contractures, and promote scar management through the use of splints and pressure garments [33]. For small-area partial-thickness burns, a pediatric randomized clinical trial found that adjunctive negative pressure wound therapy accelerated re-epithelialization (median 8 vs 10 days; ~22% faster), enabled earlier dressing autonomy, and reduced scar-management referrals [34].

Pediatric burn patients present with distinct challenges that extend beyond the trauma. Children have a higher surface area-to-body mass ratio, placing them at greater risk for the instantaneous loss of fluid and heat, consequently resulting in hypothermia and shock [28]. Furthermore, their continued growth and development processes have long-term consequences, including contractures that limit movement and affect the function of the growth plates [35]. Moreover, the psychological impact of burn injury on children is significant and tends to result in heightened anxiety, fear, and body image perception disturbance. Holistic rehabilitation emphasizes effective pain management and the contributions of numerous specialties, including social rehabilitation interventions and psychological counseling [29]. Incorporating these aspects can lower the probability of acute distress and the development of chronic pain syndromes [31]. The management of children with burns must be holistic and not only treat the acute physical injury but also address the long-term functional and emotional requirements. This promotes the long-term functional and emotional well-being of pediatric burn patients [27].

Table 1 summarizes burn depth, typical appearance, pain profile, healing time, and first-line management, highlighting differences with relevance to children:

**Table 1:** Pediatric burn classification & first-line management (quick reference) Ref no: [27–33,35]

Burn depth	Layers involved	Typical appearance	Pain	Expected healing	First-line Management Highlights
Superficial (1°)	Epidermis	Erythematous, dry, blanching	Tender	~3–7 days	Cool compress, emollients; analgesia; no routine antibiotics
Partial-thickness (2°, superficial)	Epidermis + superficial dermis	Moist, blistering	Very painful	~1–3 weeks	Gentle debridement, topical antimicrobial, non-adherent dressings; close follow-up; consider NPWT as an adjunct for select small-area burns
Partial-thickness (2°, deep)	Deeper dermis	Pale/waxy, sluggish capillary refill	Variable	≥3 weeks; ↑scar risk	Specialist referral; consider early excision/grafting if progression
Full-thickness (3°)	Entire dermis ± subcutis	Leathery/charred; insensate	Minimal	Requires surgery	Early surgical consultation, excision and grafting; multimodal analgesia and rehab

Role of AI in Burn Diagnosis

AI has emerged as a transformative tool in the diagnosis of burn injuries, particularly through the development of AI-based imaging and burn classification systems. These technologies, often powered by machine learning (ML) and deep learning (DL) algorithms, enable automated analysis of burn wound images; early studies in burns report moderate-to-high diagnostic performance, with multispectral-AI and clinical-photo models showing promising accuracy in feasibility and pilot evaluations [7,8]. Advances in noninvasive techniques may allow for early interventions and improve patient outcomes. This is particularly important in pediatric cases, as accurate and early assessment of burns will lessen the challenges associated with development and pain management. Computer vision, a broader field within AI that focuses on enabling machines to interpret and process visual information, plays a central role in this advancement, by detecting subtle variations in color, texture, and wound morphology, computer vision systems can distinguish between different burn severities can improve consistency compared with unaided visual-tactile exam, which has ~67% accuracy for partial-thickness burns; adjunct tools such as LDI outperform clinical exam in children, and AI systems seek similar standardization [4,7,36]. Convolutional neural networks (CNNs) are designed to simulate the way human vision processes images and have demonstrated high proficiency in recognizing complex patterns in wound appearance that correlate with underlying tissue damage. Studies have shown that CNN-based models have been trained to differentiate superficial, partial-thickness, and full-thickness patterns on burn images; recent evaluations compare model outputs against LDI/biopsy or 21-day healing as references [7,8,37]. 

DL models (including U-Net, DeeplabV3+, PSPNet, and Mask R-CNN) have been successfully trained to categorize burns and estimate TBSA% involvement for both regular and irregularly shaped burn wounds [15]. Tools to reduce human TBSA% math errors, such as the validated NCHart-1, illustrate how simple decision aids can improve early estimates before advanced imaging is available [38]. This reflects the growing capability of AI to handle the complex, often unpredictable morphology of burn injuries. ML models have employed different approaches to wound segmentation (delineating the boundary between wound tissue and normal skin) and wound segmentation and tissue classification in burns (classifying different types of tissue found within the wound) [15,39]. These segmentation tasks are foundational for improving the accuracy of wound assessment and treatment planning. To train DL models effectively for burn detection, researchers have relied on large annotated datasets containing images of burn wounds with edges and severities classified by expert burn surgeons, ensuring clinical relevance and consistency [15,40]. Additionally, some algorithms have been designed to analyze wound images captured at different healing stages, providing predictive insights into wound healing trajectories and identifying cases where grafting may be necessary [41]. Collectively, these advancements highlight the potential of AI-driven tools to enhance both the diagnostic and prognostic aspects of burn care. 

Historically, the clinical evaluation of burn depth has relied on the subjective assessment of visual and tactile characteristics of a burn wound, achieving an accuracy of approximately 67% for partial-thickness burns [8,42]. AI-based approaches, particularly those employing CNNs, have demonstrated significantly higher diagnostic performance, with reported model-level performance in feasibility studies; for example, a multispectral-AI ensemble reported PPV 97% and specificity 100% for non-healing regions with sensitivity improving after day 2.5 [7], while clinical-photo AI assessments have shown comparable or better discrimination than unaided exam in early evaluations [8]. Pediatric-only datasets are also emerging [39]; in pediatric burns, an AI-assisted multispectral imaging approach showed 70% sensitivity, 100% specificity, positive predictive value of 97%, and negative predictive value of 100% [7]. Community triage models have also matched or exceeded clinical assessment baselines for depth/margins, complementing optics-focused ML reviews and non-invasive imaging syntheses [8,19,20]. Accurate burn depth assessment is critical in optimizing outcomes and preventing complications such as delayed healing, infection, and excessive scarring. Partial-thickness burns require timely and appropriate wound care to prevent progression to deeper injuries, whereas full-thickness burns often necessitate early surgical intervention. By supporting early recognition of injury severity, AI tools enhance the timing and specificity of therapeutic decisions [3]. Given the subjective nature of traditional assessments and differences in clinician experience, AI-based tools offer a standardized and reproducible method that improves diagnostic consistency, particularly in pediatric settings where prompt intervention is crucial [43]. Beyond burns, a systematic review shows that image-based AI for wound assessment has matured across multiple wound types (segmentation, area measurement, tissue classification), with pipelines readily transferable to burn care [44]. Despite ongoing challenges such as variability in study designs, model interpretability, and integration into clinical workflows, the potential of AI to transform burn diagnosis continues to expand. By providing more objective, consistent, and scalable solutions, AI can enhance clinical decision-making and significantly improve patient care outcomes.

AI in Treatment Planning/Monitoring

The use of AI supports the field of pediatric burn care by enabling the individualization of treatment plans, predicting healing timelines and complications, and monitoring the progress of healing and treatment efficacy. Data-driven decision-support systems may assist with risk stratification (e.g., graft need, infection risk) and monitoring; however, fully automated treatment-planning for pediatric burns has not been clinically validated and should be framed as a research direction [7,8,45,46]. Recent papers also detail how AI integrates with wound-healing biology, smart dressings, and biofabrication, including 3D/bioprinting and robotic control, to enable closed-loop, individualized care [47-50]. These innovations allow for more responsive and individualized care strategies.

AI has great potential within burn care by predicting timelines and healing durations. Its ability to anticipate complications such as infections or hypertrophic scarring renders it crucial to the healing process. ML models, trained on wide-ranging clinical datasets, have demonstrated important predictive capabilities that identify discernible patterns in variables like burn depth, infection markers, and patient demographics [47,51]. Healthcare teams can adjust treatment plans proactively due to their ability to predict complications early, resulting in improved outcomes and a decreased number of long-term sequelae for pediatric patients.

With wearable health technologies advancing, AI has found its place in wearable devices for treatment, allowing for continuous monitoring of wound healing [48,52]. Critical wound parameters, such as temperature, pH, and moisture levels, can be monitored using newly developed and suitably improved flexible sensors, facilitated by AI algorithms. PETAL, an AI-enabled, battery-free sensor patch, demonstrated an accuracy of 97% in distinguishing healing from non-healing wounds [53]. Initially tested in adult populations for wound care, this technology's adaptability shows promise for pediatric applications, particularly due to its non-intrusive design and adherence to the skin. These AI sensors would enable the early detection of deviations in wound healing from its typical path, allowing physicians to intervene in a timely manner before more severe complications arise.

Telemedicine is a valuable adjunct to traditional burn care, particularly for pediatric patients facing logistical barriers to frequent in-person visits. AI assists clinicians in making diagnostic and management decisions by analyzing wound images submitted through telehealth platforms, thereby enhancing remote care capabilities [54]. According to Burns et al., parents valued the convenience, lower costs, and reduced wait times concerning telemedicine visits for their children's acute conditions [55]. However, concerns about the perceived quality of care persist compared to customary visits, suggesting that a careful integration of telehealth into pediatric burn management is needed. Continuity of care can be ensured through the design of suitable AI-assisted telemedicine platforms while maintaining clinical effectiveness and patient safety [54,56,57].

Leveraging telehealth and emerging AI/ML technologies presents a significant opportunity to expand access to pediatric burn care. Frequent in-person follow-ups often present difficulties for pediatric patients and their families. In the United States, only 62.1% of children live within 50 miles of a pediatric burn center, and burn specialists may not be readily available, particularly in rural or medically underserved areas [58]. Providing patients and their parents with secure, user-friendly tools to capture and upload high-quality wound images would enable clinicians to remotely monitor healing, adjust treatments in real-time, and intervene early if signs of infection, delayed healing, or complications arise. There is also an opportunity to integrate wearable technologies with telemedicine. Advancements in wearable technologies, for example, the skin-adherent sensors and computer-vision tools, may support real-time monitoring; to date, most demonstrations are in adult or non-burn cohorts and require pediatric validation [18,48,52,53]. Approaches that integrate innovative AI tools with telehealth have the potential to significantly reduce the travel burden, minimize hospital exposure for immunocompromised children, and support more personalized pathways to recovery.

AI-enabled telemedicine and skin-adherent sensors can extend specialist care beyond burn centers, enabling more comprehensive care. Table 2 summarizes common parameters, clinical utilities, and practical considerations for remote follow-up:

**Table 2:** Telemedicine & wearables for pediatric burns (AI-assisted) ROM: Range of motion Ref no: [48,52–55,57,58]

Modality	Parameter(s)	Clinical utility	Pediatric considerations
AI-assisted teledermatology	Image triage; depth estimation; trajectory check	Reduces travel burden; enables earlier escalation when worsening; supported by recent feasibility work in pediatrics	Caregiver training for image quality; privacy/consent workflows
Skin-adherent sensor patch (battery-free)	Temperature, pH, moisture	Detects non-healing patterns; triggers alerts	Comfort, skin sensitivity, and adhesion on small/active patients
Smart dressings/bandages	Microenvironment sensing; drug delivery	Closed-loop monitoring + therapy	Size/form factor; safe materials; interoperability with portals
Home ROM tracking (vision/sensors ± VR)	Joint angle/adherence	Gamified rehabilitation: contracture prevention	Engagement/motivation; age-appropriate design/experience; caregiver dashboards

AI in Surgical Planning/Rehabilitation

AI becomes a helpful instrument as therapists mend and surgeons map pediatric burn injuries. AI technologies are improving the quality and precision of care, from graft modeling optimization to surgical outcomes prediction and rehabilitation support. Burn reconstruction requires accurate skin graft planning, particularly for pediatric patients, whose growth patterns and healing capacities can impact success. Wound size, wound depth, and patient-specific variables have informed graft coverage strategies, which are simulated using ML algorithms [8,15,59]. These algorithms have also been used to model wound areas. DL methods, notably convolutional neural networks, have demonstrated efficacy in automating burn segmentation from clinical photos and allowing surgeons to improve excision and graft placement. Furthermore, AI-driven 3D-printing/bioprinting and scaffold optimization remain experimental and outside routine pediatric burn surgery [50].

Predictive analytics is increasingly important in surgical decision-making for burn patients. AI models, trained upon historical patient data, can forecast several outcomes, such as graft take rates, infection risks, scar formation, and functional recovery. Specific decision tree algorithms have been employed to predict the success of grafts. Preoperative factors, such as patient age, burn severity, wound microbiology, and nutritional status, are important considerations [51]. Healthcare teams identify high-risk cases quite early by using predictive modeling. Thus, they thoroughly modify perioperative strategies, improving surgical safety and long-term results [60].

Rehabilitation plays a critical role in helping pediatric burn survivors recover, restore mobility, prevent contractures, and improve their quality of life. AI applications are beginning to transform traditional physiotherapy approaches by personalizing rehabilitation programs based on real-time patient feedback [61]. Wearable sensors embedded with AI capabilities can monitor joint range of motion, muscle activation, and exercise adherence, providing data-driven insights for therapists. AI-powered virtual reality (VR) platforms are also being introduced to improve engagement throughout physiotherapy sessions. Via these platforms, pediatric patients complete painful range-of-motion exercises in engaging, gamified environments [62]. Predictive rehabilitation models also emerge, allowing clinicians to forecast functional recovery trajectories and adjust therapy intensity accordingly. Pediatric burn teams can care with greater precision, in a more personalized manner, and in a responsive way, which optimizes surgical outcomes and functional recovery through the integration of AI into surgical planning and rehabilitation.

AI tools now support multiple decision points, from triage image classification to rehabilitation monitoring. Table 3 maps representative tasks, model families, training inputs, and example outputs across the pediatric burn care continuum:

**Table 3:** Where AI fits in pediatric burn care TBSA: Total body surface area; ML/DL: Machine Learning/Deep Learning Ref no: [7,8,15,22–25,37–41,47,48,51–53]

Care phase	Clinical question	AI task/type	Typical inputs	Example outputs/uses
Triage/ED	What depth? How much TBSA%?	Image classification & semantic segmentation (CNNs; U-Net/DeepLab/Mask R-CNN variants)	Smartphone/clinic photos; clinician-captured images	Depth class (1°/2°/3°), pixel-level masks, TBSA% estimates; simple aids like NCHart-1 reduce human TBSA% math errors.
Early course	Will this wound need grafting?	Prognosis modeling (ML/DL; survival/gradient boosting)	Depth, TBSA%, location, age, vitals, labs	Risk scores for delayed healing or grafting; suggested follow-up interval
Inpatient/OR	How to plan graft?	Computer vision + 3D modeling	Wound contours, body-site geometry	Graft sizing, donor-site planning, virtual simulation of tension/elasticity
Post-op	Infection risk next 72h?	Predictive analytics (trees/GBMs)	Microbiology, temps, dressings, comorbidities	Early infection alerts; antibiotic stewardship prompts
Rehab/home	Is the wound healing normally?	Wearable sensing + anomaly detection	Temperature, pH, moisture time-series	Healing vs non-healing classification; adherence prompts; tele-visit triage

Ethical, Legal, and Social Implications (ELSI)

Privacy and consent for minors: Pediatric image capture requires explicit parental permission and age-appropriate assent. Images should be de-identified at source, stored with role-based access, and if reused for model training, covered by transparent consent and governance [63-65].

Algorithmic bias and equity: Training sets skewed by skin tone, age mix, and care setting can propagate errors and inequities; audits should track performance across Fitzpatrick types and demographic subgroups [66,67]. Clinical safety & validation: Before deployment of AI tools, external validation against accepted comparators (e.g., LDI, 21-day healing) is required, with prospective monitoring for drift, with clear human-in-the-loop oversight and fallback pathways [37,46,65].

Data governance and accountability: Specify data controllers, model provenance/versioning, update cadence, and clinician responsibility for final decisions; document failures and corrections in a model registry accessible to the clinical team [63,65].

Implementation and family communication: Provide plain-language statements for families on how images/data are used, expected benefits/limits of AI, and how to opt out, especially for remote triage/telemedicine [68].

## Conclusions

The use of AI tools, including ML, DL, and generative AI, and their integration with telemedicine and wearable technologies, represents a promising and developing frontier in pediatric burn care. AI technologies have the potential to significantly enhance how clinicians diagnose, monitor, and treat burn injuries in children by improving diagnostic speed and accuracy, predicting complications, personalizing care, and expanding access to expert burn care in resource-limited areas. To fully realize these benefits, continued development of these tools is essential. Advancing the design of wearable technologies tailored to pediatric patients, continued expansion of pediatric burn wound datasets, and improvement of AI algorithms through clinical validation are necessary before implementation in patient care settings. Addressing both the technical limitations and ethical challenges of AI tools will be crucial to ensuring that these innovations enhance the diagnosis, treatment, and responsible long-term management of pediatric burns.

## References

[1] Nassar JY, Al Qurashi AA, Albalawi IA (2023). Pediatric burns: A systematic review and meta-analysis on epidemiology, gender distribution, risk factors, management, and outcomes in emergency departments. Cureus.

[2] Ciornei B, David VL, Popescu D, Boia ES (2023). Pain management in pediatric burns: A review of the science behind it. Glob Health Epidemiol Genom.

[3] Stewart S, Juang D, Aguayo P (2022). Pediatric burn review. Semin Pediatr Surg.

[4] Holland AJA, Martin HCO, Cass DT (2002). Laser doppler imaging prediction of burn wound outcome in children. Burns.

[5] Mill J, Cuttle L, Harkin DG, Kravchuk O, Kimble RM (2009). Laser doppler imaging in a paediatric burns population. Burns.

[6] Khatib M, Jabir S, Fitzgerald O'Connor E, Philp B (2014). A systematic review of the evolution of laser doppler techniques in burn depth assessment. Plast Surg Int.

[7] Thatcher JE, Yi F, Nussbaum AE (2023). Clinical investigation of a rapid non-invasive multispectral imaging device utilizing an artificial intelligence algorithm for improved burn assessment. J Burn Care Res.

[8] Lee JJ, Abdolahnejad M, Morzycki A (2025). Comparing artificial intelligence guided image assessment to current methods of burn assessment. J Burn Care Res.

[9] Pape SA, Skouras CA, Byrne PO (2001). An audit of the use of laser doppler imaging (LDI) in the assessment of burns of intermediate depth. Burns.

[10] Hoeksema H, Van de Sijpe K, Tondu T, Hamdi M, Van Landuyt K, Blondeel P, Monstrey S (2009). Accuracy of early burn depth assessment by laser doppler imaging on different days post burn. Burns.

[11] Monstrey S, Hoeksema H, Verbelen J, Pirayesh A, Blondeel P (2008). Assessment of burn depth and burn wound healing potential. Burns.

[12] Cubison TC, Pape SA, Parkhouse N (2006). Evidence for the link between healing time and the development of hypertrophic scars (HTS) in paediatric burns due to scald injury. Burns.

[13] Chipp E, Charles L, Thomas C, Whiting K, Moiemen N, Wilson Y (2017). A prospective study of time to healing and hypertrophic scarring in paediatric burns: Every day counts. Burns Trauma.

[14] Romanowski KS, Palmieri TL (2017). Pediatric burn resuscitation: Past, present, and future. Burns Trauma.

[15] Chang CW, Lai F, Christian M (2021). Deep learning-assisted burn wound diagnosis: Diagnostic model development study. JMIR Med Inform.

[16] Esteva A, Kuprel B, Novoa RA, Ko J, Swetter SM, Blau HM, Thrun S (2017). Dermatologist-level classification of skin cancer with deep neural networks. Nature.

[17] Taib BG, Karwath A, Wensley K, Minku L, Gkoutos GV, Moiemen N (2023). Artificial intelligence in the management and treatment of burns: A systematic review and meta-analyses. J Plast Reconstr Aesthet Surg.

[18] Vo DK, Trinh KT (2025). Advances in wearable biosensors for wound healing and infection monitoring. Biosensors (Basel).

[19] Wilson RH, Rowland R, Kennedy GT (2024). Review of machine learning for optical imaging of burn wound severity assessment. J Biomed Opt.

[20] Li H, Bu Q, Shi X, Xu X, Li J (2024). Non-invasive medical imaging technology for the diagnosis of burn depth. Int Wound J.

[21] Tan P, Ravulapalli K, Lewis CJ (2025). A systematic review of advances in the use of spectral imaging in burn depth assessment. Burns.

[22] Boissin C, Laflamme L, Fransén J (2023). Development and evaluation of deep learning algorithms for assessment of acute burns and the need for surgery. Sci Rep.

[23] Xu X, Bu Q, Xie J, Li H, Xu F, Li J (2024). On-site burn severity assessment using smartphone-captured color burn wound images. Comput Biol Med.

[24] Carter JE, Shupp JW, Phelan HA, Hickerson W, Cockerell CJ, DiMaio M, Holmes JH 4th (2025). Refinement of an artificial intelligence algorithm for enhanced burn wound depth assessment using multispectral imaging: An expanded proof of concept study. J Burn Care Res.

[25] Lindert J, Straube T, Larsen B, Siebert J, Liodaki E, Tafazzoli-Lari K, Wünsch L (2024). An optical tomography-based score to assess pediatric hand burns. Eur Burn J.

[26] Wang R, Zhao J, Zhang Z, Cao C, Zhang Y, Mao Y (2020). Diagnostic accuracy of laser Doppler imaging for the assessment of burn depth: A meta-analysis and systematic review. J Burn Care Res.

[27] Żwierełło W, Piorun K, Skórka-Majewicz M, Maruszewska A, Antoniewski J, Gutowska I (2023). Burns: Classification, pathophysiology, and treatment: A review. Int J Mol Sci.

[28] Krishnamoorthy V, Ramaiah R, Bhananker SM (2012). Pediatric burn injuries. Int J Crit Illn Inj Sci.

[29] Woolard A, Hill NT, McQueen M (2021). The psychological impact of paediatric burn injuries: A systematic review. BMC Public Health.

[30] Cartotto R (2017). Topical antimicrobial agents for pediatric burns. Burns Trauma.

[31] Gandhi M, Thomson C, Lord D, Enoch S (2010). Management of pain in children with burns. Int J Pediatr.

[32] Suman A, Owen J (2020). Update on the management of burns in paediatrics. BJA Educ.

[33] Ohgi S, Gu S (2013). Pediatric burn rehabilitation: Philosophy and strategies. Burns Trauma.

[34] Frear CC, Cuttle L, McPhail SM, Chatfield MD, Kimble RM, Griffin BR (2020). Randomized clinical trial of negative pressure wound therapy as an adjunctive treatment for small-area thermal burns in children. Br J Surg.

[35] Yelvington M, Whitehead C, Turgeon L (2023). Special considerations for pediatric burn injuries. Phys Med Rehabil Clin N Am.

[36] Jeschke MG, van Baar ME, Choudhry MA, Chung KK, Gibran NS, Logsetty S (2020). Burn injury. Nat Rev Dis Primers.

[37] Cirillo MD, Mirdell R, Sjöberg F, Pham TD (2019). Time-independent prediction of burn depth using deep convolutional neural networks. J Burn Care Res.

[38] Ray WC, Rajab A, Alexander H (2022). A 1% TBSA chart reduces math errors while retaining acceptable first-estimate accuracy. J Burn Care Res.

[39] Li X, Liu Z, Liu L (2025). Pediatric BurnNet: Robust multi-class segmentation and severity recognition under real-world imaging conditions. SAGE Open Med.

[40] Rangaiah PKB, Kumar BPP, Augustine R (2025). Improving burn diagnosis in medical image retrieval from grafting burn samples using B-coefficients and the CLAHE algorithm. Biomed Signal Process Control.

[41] Rangaiah PK, Kumar BP, Huss F, Augustine R (2025). Precision diagnosis of burn injuries using imaging and predictive modeling for clinical applications. Sci Rep.

[42] Devgan L, Bhat S, Aylward S, Spence RJ (2006). Modalities for the assessment of burn wound depth. J Burns Wounds.

[43] Marcaccini G, Seth I, Lim B (2025). Management of burns: Multi-center assessment comparing AI models and experienced plastic surgeons. J Clin Med.

[44] Anisuzzaman DM, Wang C, Rostami B, Gopalakrishnan S, Niezgoda J, Yu Z (2022). Image-based artificial intelligence in wound assessment: A systematic review. Adv Wound Care (New Rochelle).

[45] E Moura FS, Amin K, Ekwobi C (2021). Artificial intelligence in the management and treatment of burns: A systematic review. Burns Trauma.

[46] Topol EJ (2019). High-performance medicine: the convergence of human and artificial intelligence. Nat Med.

[47] Jeraj ZR, Zameer Z (2025). AI-enhanced bioactive 3D-printed scaffolds for tissue regeneration: innovations in healing and functional additives. J Comput Biomed Informatics.

[48] Mostafalu P, Tamayol A, Rahimi R (2018). Smart bandage for monitoring and treatment of chronic wounds. Small.

[49] Zhu G, Fu Z, Guo G (2025). Applications and prospects of artificial intelligence in wound healing. Regenesis Repair Rehabilitation.

[50] Zhang Z, Zhou X, Fang Y, Xiong Z, Zhang T (2025). AI-driven 3D bioprinting for regenerative medicine: From bench to bedside. Bioact Mater.

[51] Liang H, Tsui BY, Ni H (2019). Evaluation and accurate diagnoses of pediatric diseases using artificial intelligence. Nat Med.

[52] Heikenfeld J, Jajack A, Rogers J (2018). Wearable sensors: Modalities, challenges, and prospects. Lab Chip.

[53] Zheng XT, Yang Z, Sutarlie L (2023). Battery-free and AI-enabled multiplexed sensor patches for wound monitoring. Sci Adv.

[54] Krick T, Huter K, Domhoff D, Schmidt A, Rothgang H, Wolf-Ostermann K (2019). Digital technology and nursing care: A scoping review on acceptance, effectiveness and efficiency studies of informal and formal care technologies. BMC Health Serv Res.

[55] Burns SK, Krishnamurti T, Doan TT (2024). Parent perceptions of telemedicine for acute pediatric respiratory tract infections: Sequential mixed methods study. JMIR Pediatr Parent.

[56] Esberk T, Das K (2023). Telemedicine for the management of pediatric burn patients admitted to emergency services. J Telemed Telecare.

[57] Frederick AB, Gavrilova Y, Koren N (2025). A randomized feasibility study of virtual and face-to-face care using a novel mobile health solution for outpatient pediatric burns. J Burn Care Res.

[58] Dougherty JM, Blake ES, Rittle CJ, Fan Z, Hunter MA, Hemmila MR, Sangji NF (2025). Challenges in geographic access to specialized pediatric burn care in the United States. J Burn Care Res.

[59] Sun X, Zhang Y, Ma C, Yuan Q, Wang X, Wan H, Wang P (2021). A review of recent advances in flexible wearable sensors for wound detection based on optical and electrical sensing. Biosensors (Basel).

[60] Brusselaers N, Monstrey S, Vogelaers D, Hoste E, Blot S (2010). Severe burn injury in Europe: A systematic review of the incidence, etiology, morbidity, and mortality. Crit Care.

[61] Aldhahi MI, Alorainy AI, Abuzaid MM, Gareeballah A, Alsubaie NF, Alshamary AS, Hamd ZY (2025). Adoption of artificial intelligence in rehabilitation: Perceptions, knowledge, and challenges among healthcare providers. Healthcare (Basel).

[62] Jeffs D, Dorman D, Brown S (2014). Effect of virtual reality on adolescent pain during burn wound care. J Burn Care Res.

[63] Vayena E, Blasimme A (2018). Health research with big data: Time for systemic oversight. J Law Med Ethics.

[64] Shabani M, Thorogood A, Borry P (2016). Who should have access to genomic data and how should they be held accountable? Perspectives of data access committee members and experts. Eur J Hum Genet.

[65] Gerke S, Minssen T, Cohen G (2020). Ethical and legal challenges of artificial intelligence-driven healthcare. Elsevier.

[66] Buolamwini J, Gebru T (2018). Gender shades: Intersectional accuracy disparities commercial gender classification. Proc Mach Learn Res.

[67] Obermeyer Z, Powers B, Vogeli C, Mullainathan S (2019). Dissecting racial bias in an algorithm used to manage the health of populations. Science.

[68] Alfeir NM (2024). Dimensions of artificial intelligence on family communication. Front Artif Intell.

